# Real-Time Model Predictive Control of Lignin Properties Using an Accelerated kMC Framework with Artificial Neural Networks

**DOI:** 10.1021/acs.iecr.4c02918

**Published:** 2024-11-19

**Authors:** Juhyeon Kim, Jiae Ryu, Qiang Yang, Chang Geun Yoo, Joseph Sang-II Kwon

**Affiliations:** † Artie McFerrin Department of Chemical Engineering, Texas A&M University, College Station, Texas 77845, United States; ‡ Texas A&M Energy Institute, Texas A&M University, College Station, Texas 77845, United States; § Department of Chemical Engineering, State University of New York College of Environmental Science and Forestry, Syracuse, New York 13210, United States; ∥ School of Packaging, Michigan State University, East Lansing, Michigan 48824, United States

## Abstract

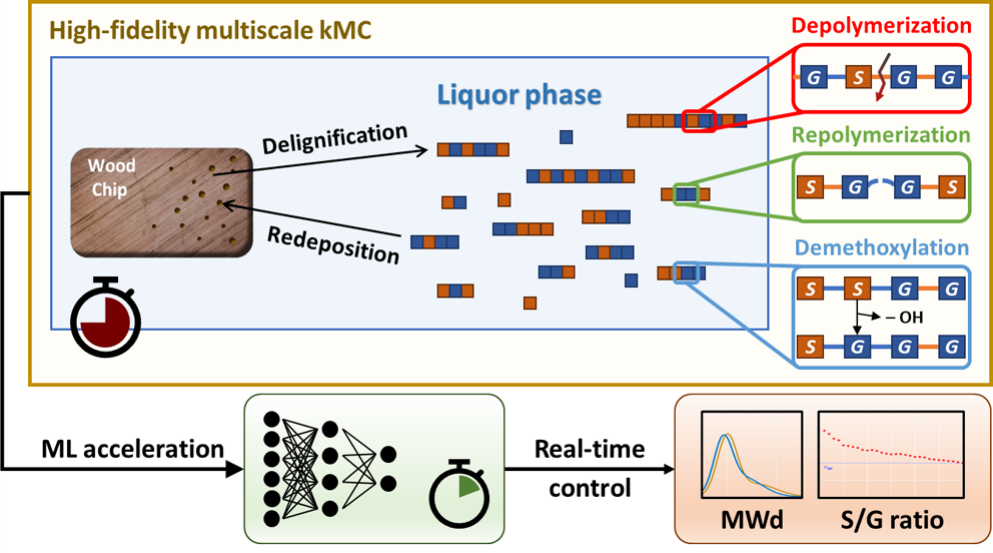

While lignin has garnered significant research interest for its abundance and versatility, its complicated structure poses a challenge to understanding its underlying reaction kinetics and optimizing various lignin characteristics. In this regard, mathematical models, especially the multiscale kinetic Monte Carlo (kMC) method, have been devised to offer a precise analysis of fractionation kinetics and lignin properties. The kMC model effectively handles the simulation of all particles within the system by calculating reaction rates between species and generating a rate-based probability distribution. Then, it selects a reaction to execute based on this distribution. However, due to the vast number of lignin polymers involved in the reactions, the rate calculation step becomes a computational bottleneck, limiting the model’s applicability in real-time control scenarios. To address this, the machine learning (ML) technique is integrated into the existing kMC framework. By training an artificial neural network (ANN) on the kMC data sets, we predict the probability distributions instead of repeatedly calculating them over time. Subsequently, the resulting ANN-accelerated multiscale kMC (AA-M-kMC) model is incorporated into a model predictive controller (MPC), facilitating real-time control of intricate lignin properties. This innovative approach effectively reduces the computational burden of kMC and advances lignin processing methods.

## 1 Introduction

Lignin, a complex and heterogeneous biopolymer, holds significant promise for a sustainable future due to its abundance and numerous potential applications in biofuels, chemicals, and materials.^1−4^ However, its successful utilization remains obscure since lignin processing faces significant obstacles stemming from its intricate structure.^5−7^ To tackle this, numerous experimental studies have aimed to enhance fractionation techniques by developing novel solvents^8−11^ or catalysts.^12−15^ Recently, in line with these efforts, various modeling techniques have also emerged to study the underlying kinetics of lignin reactions. Such endeavors include the lumped kinetic model^16^ and the population balance model^17^ to track lignin properties, including monomer yields^18,19^ and the average molecular weight of the resulting lignin.^20,21^ However, these works primarily focused on the depolymerization reactions. Despite their valuable insight into lignin depolymerization, models for studying the full fractionation processes and controlling the lignin properties are still limited.

To overcome this, multiscale modeling approaches have been developed to provide a comprehensive understanding of the overall fractionation process,^22−25^ including lignin dissolution from the woody biomass feedstocks (i.e., delignification) and their depolymerization. In these studies, the kinetic Monte Carlo (kMC) method has offered a numerical approach to solving the chemical master equation for complicated systems.^26−28^ The kMC provides detailed insights throughout the microscopic depolymerization reactions, and it is integrated with the macroscopic delignification model, completing global lignin mass balance and multiscale model structure for the whole fractionation system. Such high-fidelity models can be utilized in control approaches to determine the optimal recipe for achieving diverse lignocellulosic material properties as desired.^29,30^ However, the kMC models are computationally expensive due to their stochastic algorithm and detailed system description. Specifically, calculating reaction rates is the primary computational bottleneck in the kMC framework, as it involves determining the rates of numerous possible reactions for a vast number of chemical species involved in the reaction. In terms of model-based control, while a model predictive controller (MPC) is considered a viable strategy to address this problem, integrating multiscale models has not yet been considered feasible for real-time control of the lignin properties. If the embedded model is computationally demanding, obtaining a real-time solution within each sampling time becomes challenging, leading to poor control performance and negative impacts on product quality.

Recent advances have demonstrated the utility of machine learning (ML) techniques in multiscale systems modeling. These approaches have been used to capture complex dynamics across different scales and integrate them into control systems for chemical processes.^31−33^ ML models, particularly data-driven approaches, have been integrated into nonlinear MPCs for real-time process control.^34^ Moreover, the Koopman operator theory and the Operable Adaptive Sparse Identification of Systems (OASIS) framework have shown potential in representing nonlinear dynamics.^35,36^ However, these methods often involve the linearization of nonlinear systems or require extensive data preprocessing and parameter tuning, which may limit their application in some scenarios.

Inspired by this challenge, we developed an artificial neural network (ANN)-accelerated kMC framework, which offers a unique solution by directly predicting reaction rates, reducing computational demands without compromising accuracy. In this approach, the kMC algorithm calculates reaction rates at each time step based on the current system configuration, but the computationally expensive rate calculation step is replaced by an ANN trained on kMC-generated data sets. This substitution retains the detailed tracking capability of the kMC model, as all other components, including system updates and mechanisms, remain intact. The outputs of the ANN-kMC model are validated against the experiments, demonstrating its effectiveness in accelerating computations. Also, the model accuracy remains similar to the traditional kMC, while the computation time is reduced to less than 1%. Subsequently, the developed ANN-accelerated multiscale kMC (AA-M-kMC) model is seamlessly integrated into the MPC, facilitating real-time control of the biomass fractionation processes and optimizing lignin properties. The results confirm the outstanding performance of the accelerated MPC based on the AA-M-kMC model, showing precise control of key variables such as the molecular weight distribution (MWd) and the monomeric ratio.

The rest of this article is structured as follows: Section 2 discusses the mathematical formulation of the multiscale kMC model, followed by the ANN training process and integrating the ANN into the kMC algorithm. Subsequently, the open-loop simulation results using the AA-M-kMC model are also provided. In Section 3, an MPC is designed to regulate the lignin properties. Subsequently, its performance and real-time control capability are demonstrated. Then, we conclude with its significance and remarks in Section 4.

## 2 Model Formulation and Open-Loop Simulation

### 2.1 Preliminaries

**Figure 1:**
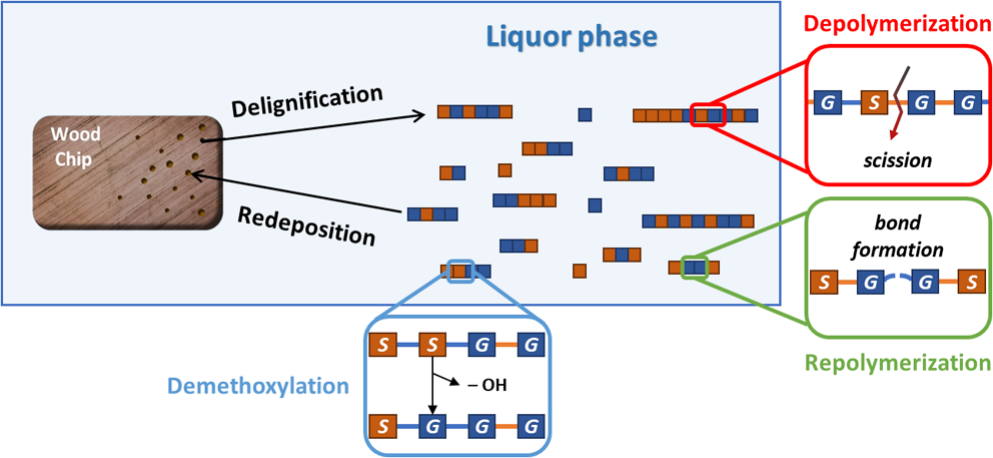
Brief illustration of the fractionation process. Two different monolignols and bonds are in distinct colors. The orange bonds stand for cleavable β–O–4 bonds, while the blue bonds are for any other uncleavable bonds.

A schematic diagram for the fractionation process is shown in Figure 1. The process involves two phases: the chip phase and the liquor phase. The reactions can be classified into two categories: slow delignification and redeposition between the two phases (macroscopic), and fast de/repolymerization and demethoxylation within the liquor phase (microscopic). It is important to note that the reactions in these two categories occur on distinct length scales, leading to significant differences in their rates. If the kMC were to evaluate all the probabilities simultaneously, the slower macroscopic reactions have relatively lower probabilities of being selected. This does not mean that slower reactions never occur, but they require much longer simulation times to be observed at the same frequency as the faster reactions. On the other hand, a layered kMC can appropriately account for these time scale differences, ensuring that both fast and slow reactions are accurately modeled according to their respective time scales, while preventing the slower reaction from being overlooked.

Therefore, a two-layer kMC model, comprising a delignification and a depolymerization layer, was used in this work. As a multiscale model, these two layers are updated simultaneously and interact with each other at every time step, providing rich insights into the reacting system. However, the kMC algorithm calculates every reaction rate for all species for each time step, demanding significant computational resources. Hence, to substitute the rate calculating step, ANN training is performed based on the kMC model outputs. In this ANN-kMC framework, the resulting ANN predicts the rates, the kMC algorithm executes a reaction using the ANN prediction, and the system information is updated. The proposed ANN-kMC does not compromise simulation accuracy, while the CPU time is significantly reduced to around 1% of its original time. Based on these results, the ANN-kMC was directly incorporated into the MPC to regulate lignin MWd and S/G ratio, completing the AA-M-kMC model. Each component will be described in the following subsections.

### 2.2 kMC Framework Description

The properties of the Aspen wood chip used in this study are listed below:

- Prepared chip size: 1.0 mm
- Lignin content: 19.6% of the dry chip weight
- Lignin initial MW: 13 kDa
- Monolignol MW: 0.227 (S) and 0.179 (G) kDa
- Initial S/G ratio: 1.76
- β–O–4 bond content: 84%

In this work, only syringyl (S) and guaiacyl (G) monomeric units are considered, as they constitute the most significant portion of the Aspen lignin. Also, the S/G ratio is defined as the number of S units divided by the number of G units. Using the above information, the wood chip is first configured. The S and G units are randomly assigned to build a 13 kDa lignin chain. This process is repeated until the total lignin mass reaches the specified amount, ensuring the overall S/G ratio is 1.76. Additionally, these monomers are bonded by different types of bonds, including carbon–oxygen (C–O) and carbon–carbon (C–C). Among them, since β–O–4 bonds constitute the majority, it is assumed that all C–O bonds in our system are β–O–4.

#### 2.2.1 Delignification Layer

The delignification reaction releases a lignin chain from the chip phase to the liquor phase. Note that the dissolved lignin can also be redeposited to the chip phase. For these two reactions, the reversible first-order kinetics is proposed and their reaction rates can be expressed as follows^37^
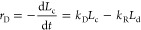
1a
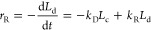
1bwhere the subscripts D and R denote delignification and redeposition, respectively. *L*_c_ and *L*_d_ stand for the lignin in the chip phase and the dissolved lignin in the liquor phase, respectively. Throughout this article, the rate constants *k* follow the Arrhenius-type equation, *k* = *A*e^–*E*_a_/*RT*^. Here, *A* and *E*_a_ are the pre-exponential factor and the activation energy barrier, respectively, *R* is the universal gas constant (0.008314 kJ/mol), and *T* is the temperature.

Additionally, the energy balance equations are also implemented. The chip phase temperature change can be represented as follows
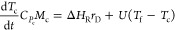
2where *T*_c_, *C*_*P*_c__, and *M*_c_ are the chip phase temperature, specific heat, and mass, respectively. In this work, *C*_*P*_c__ is expressed as a function of chip phase temperature: *C*_*P*_c__ = 0.1031 + 0.003867*T*_c_ in [kJ/kg K].^38^ Also, Δ*H*_R_ is the heat of the reaction, *U* is the overall heat transfer coefficient, and *T*_f_ is the liquor phase temperature. Since both chip and liquor phases interact, the liquor phase temperature also changes, following the equation below.
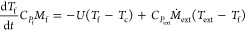
3Similar to the chip phase, *C*_*P*_f__ and *M*_f_ are the specific heat and the mass of the liquor phase. As some reactions are highly sensitive to temperature changes, the temperature will be controlled to obtain the desired lignin properties. In eq 3, the first term corresponds to the temperature change by the delignification reaction. In addition, the last term accounts for the temperature change by the external heat source, denoted by the subscript *ext*. The external heat source is applied in the open-loop simulation to maintain a constant liquor-phase temperature. Additionally, delignification and redeposition influence the liquor phase composition, and therefore, the specific heat of the liquor phase changes. Hence, a linear mixing rule is applied to estimate the specific heat of the liquor phase during the fractionation process,^39^ according to the following equation.
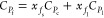
4where *x*_*f*_s__ and *x*_*f*_l__ stand for the mass fraction of the lignin molecules and solvent (PSA) in the liquor phase, and *C*_*P*_l__ is the specific heat of the pure solvent.

The delignification time resolution, i.e., the macroscopic time step, is set to be Δ*t* = 5 × 10^–4^ min. For the multiscale model to simulate the delignification over Δ*t*, the amount of dissolved lignin is calculated using eq 1a. Once the dissolved mass has accumulated to the mass of one chain, the delignification reaction is triggered, and the kMC selects the chains within the chip phases to be delignified. The redeposition process occurs in the same fashion using eq 1b. At every Δ*t*, the system configuration is updated and then delivered to the depolymerization layer.

#### 2.2.2 Depolymerization Layer

In this layer, the dissolved chains in the liquor phase can experience four reactions: depolymerization, repolymerization, demethoxylation, and null events. Depolymerization involves the scission of the given chain. Repolymerization, also known as condensation, is the head-to-tail polymerization of two arbitrary chains. Demethoxylation is a monomeric conversion where an S unit loses one methoxy group and becomes a G unit. The null event designates any other reactions that affect neither the lignin MWd nor the S/G ratio, only advancing the simulation time. Unlike the delignification and redeposition which occur across two different phases, the above four are considered microscopic.

The delignification and redeposition mass can easily be tracked in ODE form (eq 1), allowing their kinetic parameters to be determined through simple data fitting. On the other hand, to handle the complicated structure of lignin chains at the monomer level, we utilized the density functional theory (DFT) and ab initio molecular dynamics (AIMD) simulations to determine the microscopic kinetic parameters. For the selected chains and de/repolymerization sites, the activation energy barriers are calculated, considering the bond identity (e.g., S-G, S–S, G-G), system temperature, and MWd. Detailed procedures of the DFT-AIMD simulations and parameter calculations are described in our previous publication.^22^ In this work, according to their less branched nature, the lignin chains are assumed to have a linear structure.^40,41^ Additionally, the lignin MWd is assumed to follow the log-normal distribution.^42−45^ The β–O–4 bond takes the majority, and they are the most cleavable in the lignin.^46^ Therefore, it is assumed that only β–O–4 can be broken. Furthermore, the condensed bonds from repolymerization are not considered to be cleaved again.

The calculated kinetic parameters are used to determine the reaction rates for the existing chains. For any selected chain of length *N*, the depolymerization rate is expressed for each monomer–monomer bond as below
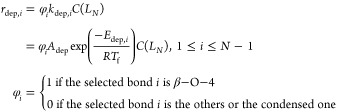
5where φ_*i*_ is a bond indicator to ensure only β–O–4 bond has the depolymerization rate and can be cleaved. Also, *C*(*L_N_*) is the concentration of the chain with its length *N. A*_dep_ is the pre-exponential factor for depolymerization, and *E*_dep,*i*_ is the activation energy barrier of the *i*-th bond within the selected chain. Note that the activation energy barrier varies by intermonolignol bonds.^22^ For repolymerization, which involves two chains, the rate is computed as follows
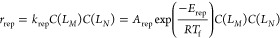
6where the subscripts *M*, *N* indicate the length of the selected lignin chains. *A*_rep_ and *E*_rep_ are the pre-exponential factor and the activation energy barrier of repolymerization. Also, the S unit has two methoxy groups that can be eliminated by the demethoxylation reaction. For the selected chain, the demethoxylation rate can be computed using the equation below
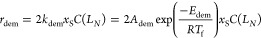
7where *x*_S_ is the molar fraction of the S unit in the selected chain. *A*_dem_ and *E*_dem_ are the pre-exponential factor and the activation energy barrier of demethoxylation. The null event does not change the kMC outputs but only advances the simulation clock. This is defined as a zeroth-order reaction, i.e., *r*_null_ = *k*_null_.

To conduct the kMC simulation, all microscopic reaction rates are summed up to *r*_sum_ at each moment. Using this, the microscopic time, δ*t*, can be calculated using the equation below^26^
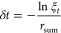
8where ξ_*t*_ ∈ [0, 1] is a randomly generated number. For every δ*t*, one microscopic reaction is selected based on the probability distribution. The execution probability is calculated by normalizing all reaction rates (eqs 5–7), and thus, a reaction with high rates has the highest probability of execution. Every reaction updates the lignin population, MWd, and the S/G ratio. Note that the microscopic reactions are significantly faster compared to the macroscopic reactions. In this model, the selection-execution process for the microscopic reactions is repeated until δ*t* accumulates to Δ*t*. Once Σδ*t* approaches Δ*t*, the simulation returns to the delignification layer, and the macroscopic events occur based on the current system configuration and eqs 1–4.

### 2.3 ANN Development

In our proposed framework, all lignin chains are tracked with respect to their own S/G compositions and chain lengths. Based on this information, the microscopic reaction rates are computed using eqs 5–7. When a microscopic event occurs, the lignin population within the liquor phase changes. Consequently, the existing kMC algorithm scans every chain and calculates the microscopic reaction rates again for every δ*t*. It should be noted that the kMC algorithm offers accurate and in-depth tracking of the simulating system, but executing one microscopic event requires exhaustive computations. This limitation poses a significant challenge in building a real-time controller based on this model.

Therefore, an ANN is trained to predict the overall distribution of reaction rates instead of calculating every individual rate, thereby reducing computational demand. The ANN inputs are the current system status, and the outputs are the microscopic reaction rates computed from the kMC model. Additionally, for ANN training, all input data were normalized to the range [0, 1] by min-max normalization. For the ANN output, the computed microscopic reaction rates are used including de/repolymerization and demethoxylation. However, each class has different levels of activation energy barriers, leading to exponential differences between the reaction rates. Therefore, to facilitate effective training, the output data were transformed using a logarithmic scale prior to normalization. In this section, a detailed ANN training procedure and its implementation into the existing kMC are discussed.

#### 2.3.1 Data Preparation

The kMC tracks diverse information including molecular weights and monolignol compositions of each lignin chain in the liquor phase. However, the primary challenge in dealing with lignin chains directly is the sheer number of chains and their structural variability, which results in high-dimensional data. In our simulations, there are thousands of lignin chains, each with unique molecular weight, bond structures, and monolignol compositions. Tracking the properties and reactions of each chain individually would not only result in substantial memory requirements but would also significantly increase the computational time due to the need to calculate the reaction rate for each chain at every time step. Therefore, there is a need for parametrization of the information on the existing lignin chains to effectively train the ANN.

First, for the existing molecular weight data to be parametrized, the Gaussian Mixture Model (GMM) is employed,^47^ and *fitgmdist* function in MATLAB is used to find the GMM. The *fitgmdist* function in MATLAB employs the Expectation-Maximization (EM) algorithm to estimate the parameters of the GMM. This iterative algorithm maximized the likelihood of the data by alternately estimating the membership of each data point to the Gaussian components (E-step) and updating the model parameters (M-step), thus effectively capturing the distribution of the data set. This approach can represent the entire lignin MWd as a few Gaussian distributions, which have finite parameters such as their respective means (μ), variances (σ), and relative weights (ω). Consequently, for the *m* Gaussian distributions, the lignin MWd information is reduced to 3*m* parameters, facilitating effective ANN training later.

***Remark 1.****Note that the depolymerization reaction breaks the lignin chains into smaller segments and there are >12,000 chains during a 30 min operation. Considering that the GMM fitting scans the MWd information for all lignin chains within the system, excessive chains require more time for the parametrization of the MWd. Therefore, to alleviate the computational cost, the 2000 lignin chains are sampled, ensuring that the distribution obtained from this reduced number of samples closely follows the original distribution. This practice reduced the GMM fitting time to 10–30%.*

**Figure 2:**
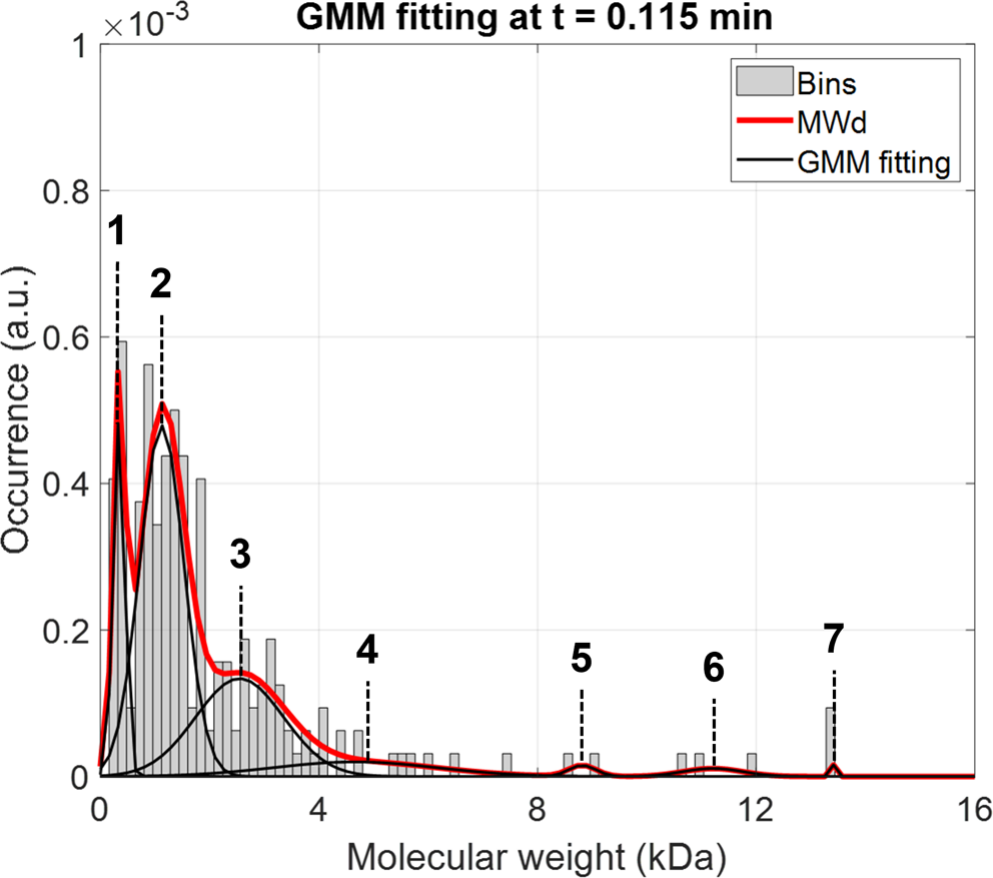
Original MWd parametrized into seven Gaussian distribution peaks.

**Figure 3:**
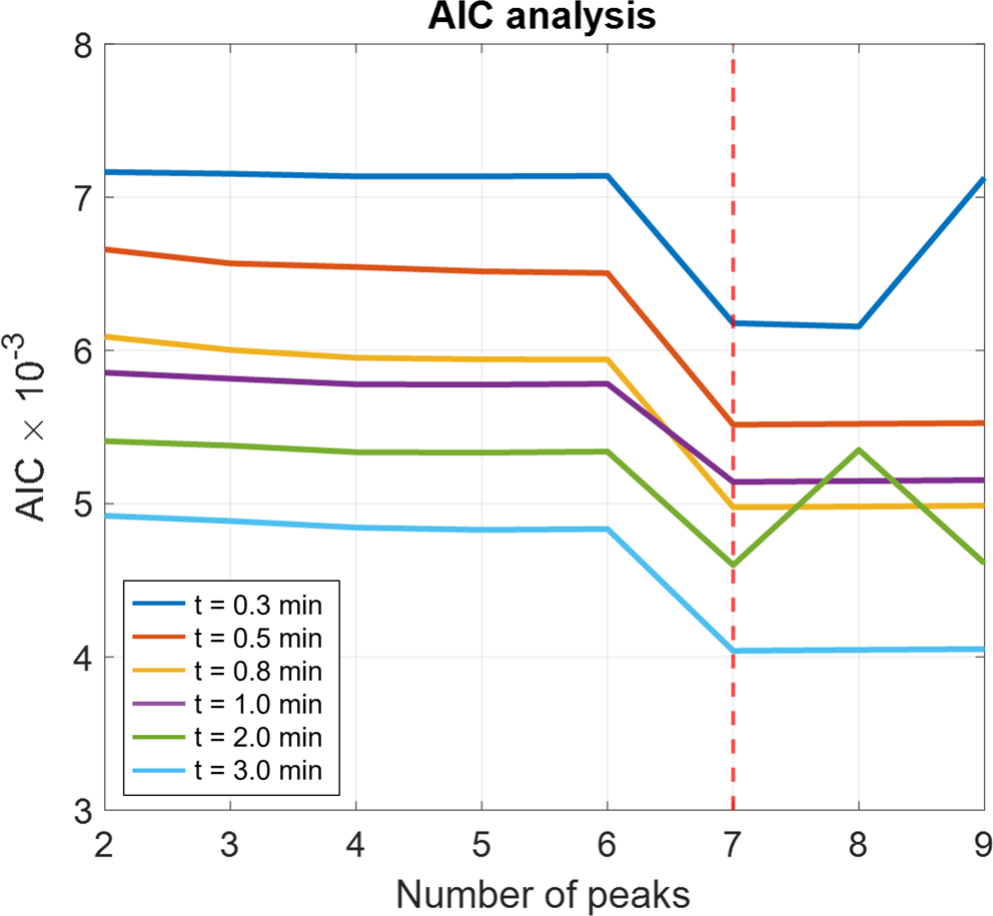
Akaike Information Criterion (AIC) evaluation to determine the optimal number of Gaussian distributions.

Subsequently, the sampled chain information is used to perform the GMM fitting. As presented in Figure 2, the lignin population is characterized well with the resulting Gaussian distributions. To determine how many peaks would best fit the original system, the Akaike Information Criterion (AIC) is used, calculated as *AIC* = −2 log *l* + 2*p*. Note that the likelihood (*l*) and the number of estimated parameters (*p*) compete against each other, resulting in the lowest AIC for the best output. According to the AICs displayed in Figure 3, seven Gaussian distributions are used to represent the current lignin MWd. Although there are diverse lengths of chains showing distinct peaks, especially in the first few minutes, note that 7 distributions are determined to be optimal. For ANN training, these 7 parameter sets (μ, σ, and ω) from these distributions are utilized instead of the entire MWd information.

Second, since the S/G ratio also influences the microscopic rates, especially the demethoxylation, the average S/G ratio is also used as a variable to reflect the effect of the monomer composition on the rates in the ANN training. Additionally, the liquor phase temperature is considered since microscopic reactions are sensitive to the temperature.

In summary, the lignin MWd and S/G ratio are parametrized to Gaussian distributions and the average ratio, respectively. Based on these data, the trained ANN predicts the distribution of microscopic reaction rates, circumventing the rate calculation for every δ*t*. The kMC algorithm then decides which reactions to execute based on the predicted rate distribution, thereby alleviating overall computational demand. The ANN training process is further explained in the next subsection.

#### 2.3.2 ANN Training

While the average of multiple kMC runs converges to certain values for MWd and S/G ratio, each kMC simulation gives different reaction trajectories due to its stochastic and probabilistic algorithm. In this regard, the kMC data sets are collected over 30 different operating conditions and used for the ANN training. To obtain the ANN training data, the MWd, S/G ratio, reactor temperature, and reaction rate data are collected every 0.025 min at 0 ≤ *t* < 1 min, and then every 1 min at *t* ≥ 1 min. This is because there are a considerably smaller number of chains in the liquor phase at that time, and they show significantly different populations for each data set.

The ANN architecture used in this work was configured with the following layers:

- Input layer: 23 nodes (including 7 sets of [μ, σ, ω], average S/G ratio, and *T*_f_).
- Hidden layers: all nodes are fully connected, and followed by a rectified linear unit (ReLU) activation function.
- Output layers: 3 nodes (including the sum of each de/repolymerization or demethoxylation).

Here, two hidden layers have 12 and 6 nodes, respectively. The Stochastic Gradient Descent with Momentum (SGDM) optimizer is used for network training and its initial learning rate is set to 0.1. The data sets are divided into 85% for training and 15% for validation. For every 15 epochs, the training process is monitored utilizing the validation data. If 15 validation tests do not lead to any improvements, the training process is terminated.

#### 2.3.3 ANN-kMC Integration

After training the ANN, the rate calculation in the kMC algorithm is replaced with the rate prediction with ANN. After the total *r*_dep_, *r*_rep_, and *r*_dem_ are predicted by the trained ANN, the selection of microscopic events is determined based on the following two-step process. The selection of de/repolymerization or demethoxylation at the current time step is weighted according to the magnitudes of the predicted total rates by ANN, ensuring that more probable events are chosen more frequently. This allows the algorithm to prioritize reactions that are more likely to occur based on the system’s current state.

Once a specific event is chosen, the algorithm selects the relevant chains and bonds for the reaction. For depolymerization or demethoxylation, a single chain is selected uniformly from the entire system. In the case of depolymerization, the activation energy barriers for each β–O–4 bond within the selected chain are evaluated, and the bond with the lowest *E*_dep,*i*_ is cleaved. For demethoxylation, one of the S monolignols in the selected chain is randomly chosen and demethoxylated by removing a methoxy group. For repolymerization, two chains are selected uniformly, and the algorithm condenses them into one longer chain. As the total reaction rates are predicted by the ANN, δ*t* can still be calculated using eq 8.

While this process simplifies the event and chain selection mechanism, it is grounded in the high-fidelity kMC simulations used to train the ANN. Since the microscopic rates are primarily affected by the lignin concentration, utilizing concentration-based selection can avoids the need for exhaustive rate calculations across all chains and bonds at each time step. This allows for a significant improvement in computational efficiency while maintaining the accuracy required for high-fidelity simulations.

**Figure 4:**
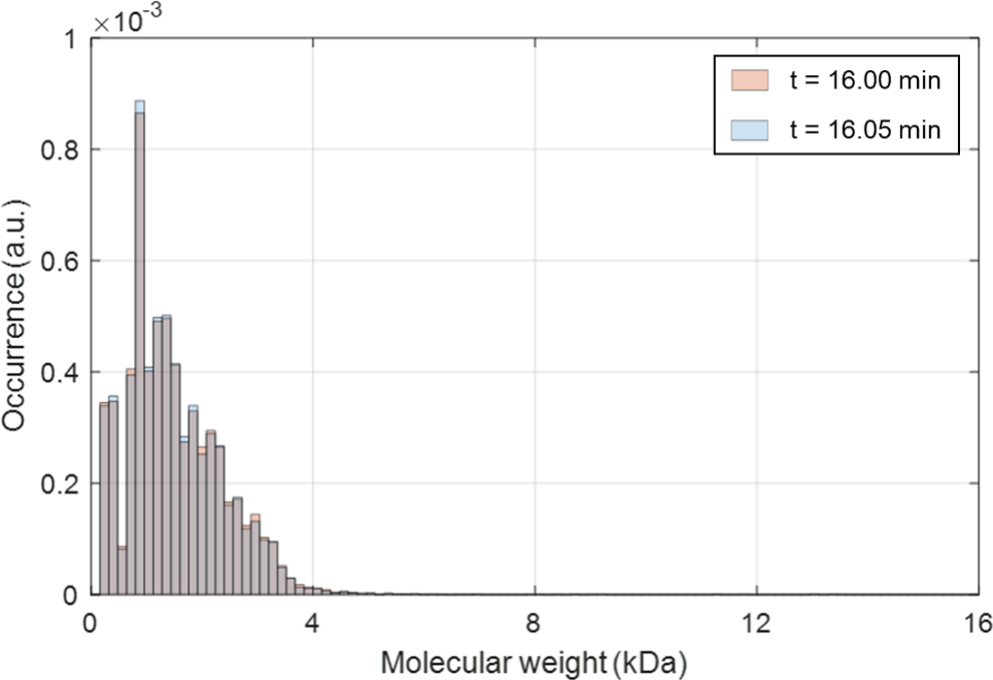
Negligible changes were observed in MWd over a period of 0.05 min, despite the execution of approximately 500 microscopic events.

This ANN-kMC serves as a surrogate kMC model, simplifying the rate calculation step. Since only the rate calculating step is substituted, the ANN-kMC retains the full advantage of the kMC simulation, such as the capability of predicting how the MWd and S/G ratio evolves over time. Note that, as shown in Figure 4, the entire distribution does not change significantly over around 500 microscopic events. Therefore, the microscopic reaction rate distribution also remains similar for a short period. In this regard, the GMM fitting and rate predictions are performed sparsely. The simulation is set to execute 500 microscopic events based on the current rate prediction and moves to the next prediction. To demonstrate its validity and feasibility, the CPU time and simulation outputs from both models are compared.

### 2.4 Open-Loop Simulation Results

**Figure 5:**
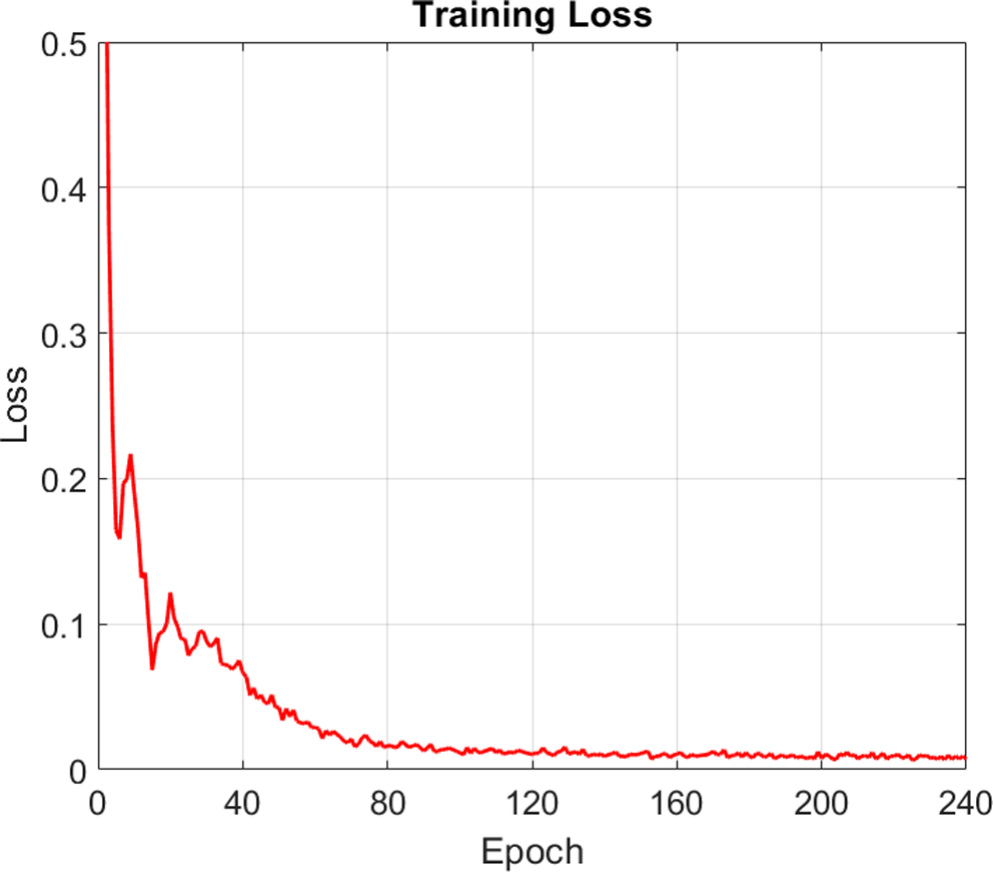
ANN training loss calculated by eq 9.

During the ANN training, the loss function of the network is given by
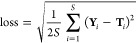
9where *S* is the number of data points, **Y** and **T** are the predicted and true values, respectively. The loss function history is shown in Figure 5, and its final value is as low as 0.0072, confirming the ANN is trained well. To further demonstrate the accuracy of the surrogate AA-M-kMC model, the results from both models are compared.

**Figure 6:**
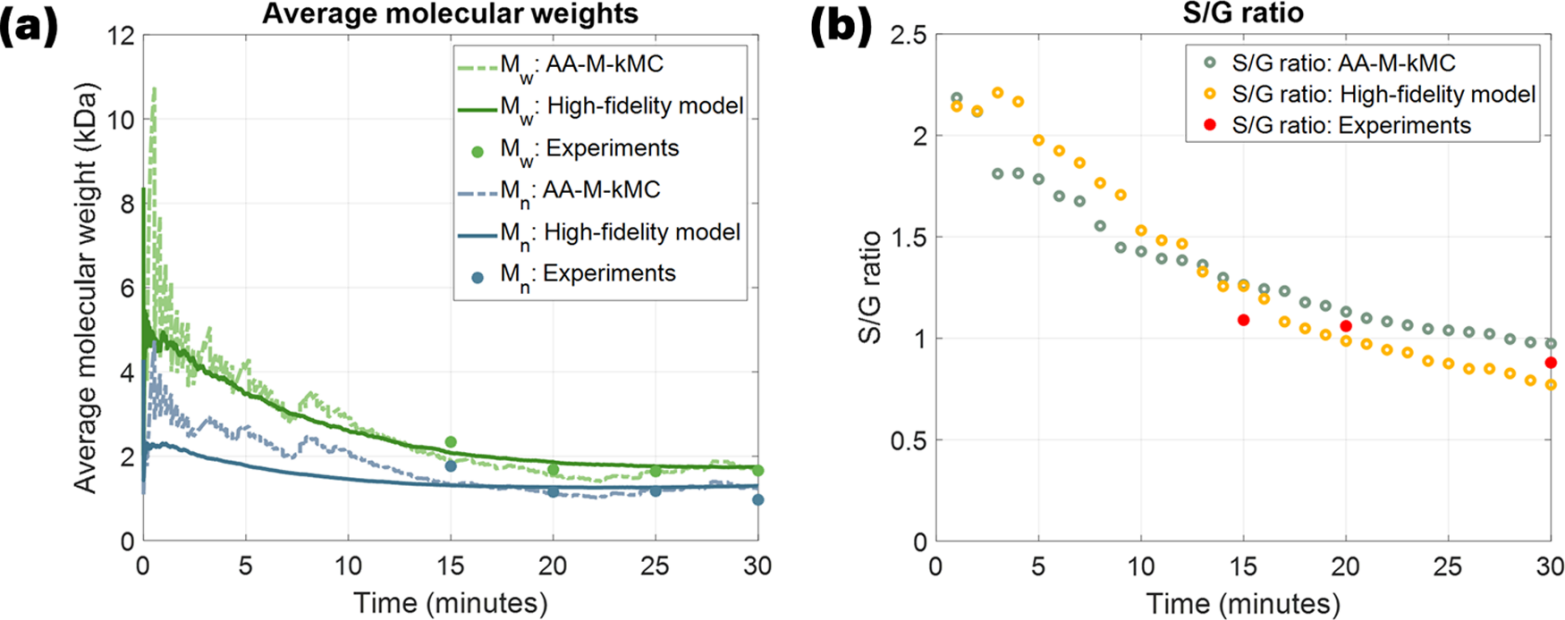
Comparison for both the high-fidelity kMC and the AA-M-kMC models: (a) average molecular weights, and (b) S/G ratio.

In Figure 6a, at 0 ≤ *t* ≤ 5, the AA-M-kMC model shows a large fluctuation due to the lumped simulation settings. The number-average (*M*_*n*_) and weight-average (*M*_w_) were calculated as defined below
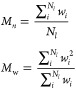
10where *N*_*l*_ is the total number of the dissolved lignin chains and *w*_*i*_ is the molecular weight of a lignin chain *i*. Given that fewer chains are dissolved in the liquor phase at the beginning, the random scission mechanism and repolymerization between a limited number of chains significantly affect the average *M*_W_, leading to an outstanding oscillation in *M*_*n*_ and *M*_w_. However, the outputs of the AA-M-kMC model converge to the experimental data after 15 min of the reaction time. As the lignin population gradually increases, the fluctuation in molecular weights is significantly alleviated and the lumped execution does not change the system aggressively. Additionally, the S/G ratio is also captured by both models and plotted in Figure 6b. In both models, the ratio initially increases, attributed to the higher population of S units, but it decreases over time due to the demethoxylation reaction. These results indicate that the number of S and G units becomes similar during fractionation. The root-mean-square errors (RMSEs) are provided in Table 1. For both the average lignin MW and the S/G ratio, the results from both models closely aligned with the experimental data.

**Table 1:** Comparison of RMSE for the High-Fidelity Model and AA-M-kMC Model

RMSE	high-fidelity model	AA-M-kMC model
*M*_*n*_ (kDa)	0.2688	0.2987
*M*_w_ (kDa)	0.2484	0.2220
S/G ratio	0.1226	0.1242

**Figure 7:**
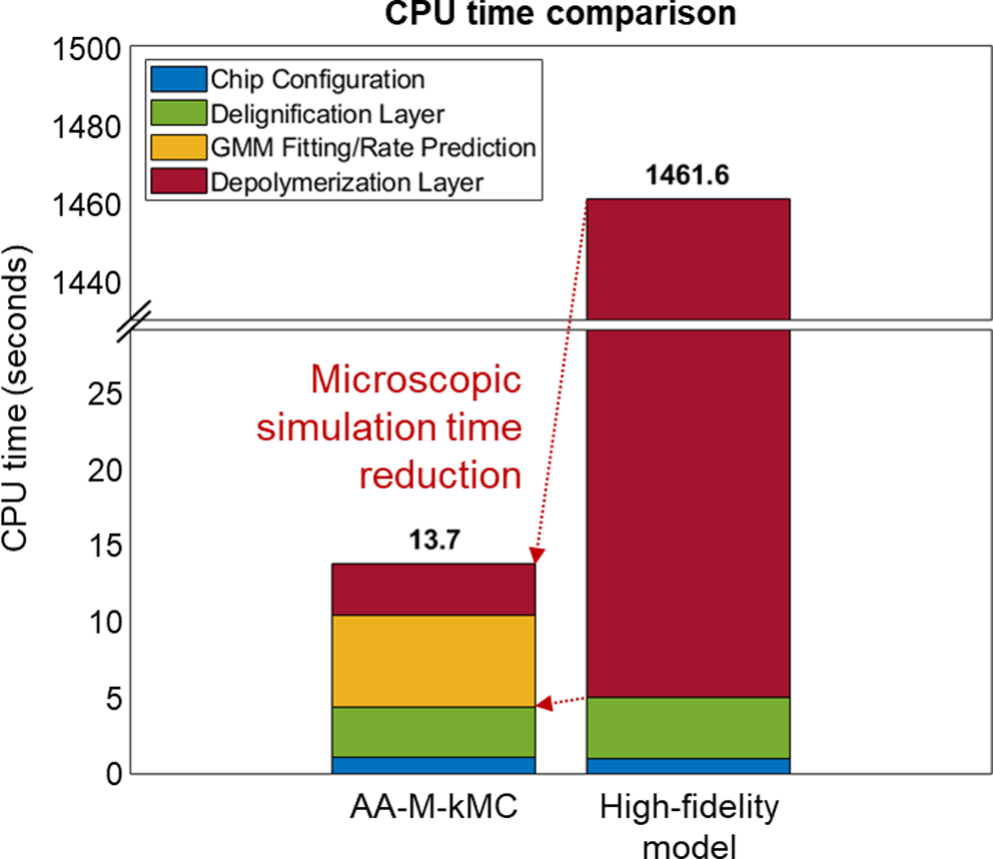
Comparison of the CPU times for each modeling approach.

Considering that both models have excellent performances, the CPU times for both models are compared to check the computational efficiency. As exhibited in Figure 7, the AA-M-kMC approach drastically reduced the computational demand. It requires substantially less simulation time, taking only about 0.94% of the time needed by the existing kMC-based model. Moreover, the main advantage of using ANN becomes evident in the depolymerization layer. In the previous kMC algorithm, the depolymerization layer conducted the rate calculation and event execution for every δ*t*, taking 1456.5 s. In contrast, the AA-M-kMC model predicts the reaction rate distribution in just 6.04 s. Also, it executes multiple microscopic events based on the predicted rates, taking 3.34 s. Therefore, for the microscopic simulation, the CPU time decreased to 0.6%, as indicated by the arrows in Figure 7.

***Remark 2.****This ANN-based acceleration technique has broad applicability across various multiscale modeling studies, especially those that include kMC simulations. By reducing computational burdens, this approach can significantly enhance the real-time control capabilities of complex systems. Similar studies have demonstrated the potential for integrating ML into multiscale simulations to optimize performance without compromising model accuracy in various applications, including thin film deposition,*(48−51)*lignocellulosic biomass,*(52,53)*and quantum dots.*(54)*This framework is not only beneficial for kMC-based models but can also be extended to other simulation-based approaches in multiscale modeling.*

## 3 MPC Design and Closed-Loop Operation Results

With the above results, the AA-M-kMC model is seamlessly implemented into the MPC framework to regulate the key properties of the resulting lignin. In this section, the MPC formulation and the closed-loop control results are presented.

### 3.1 MPC Formulation

**Figure 8:**
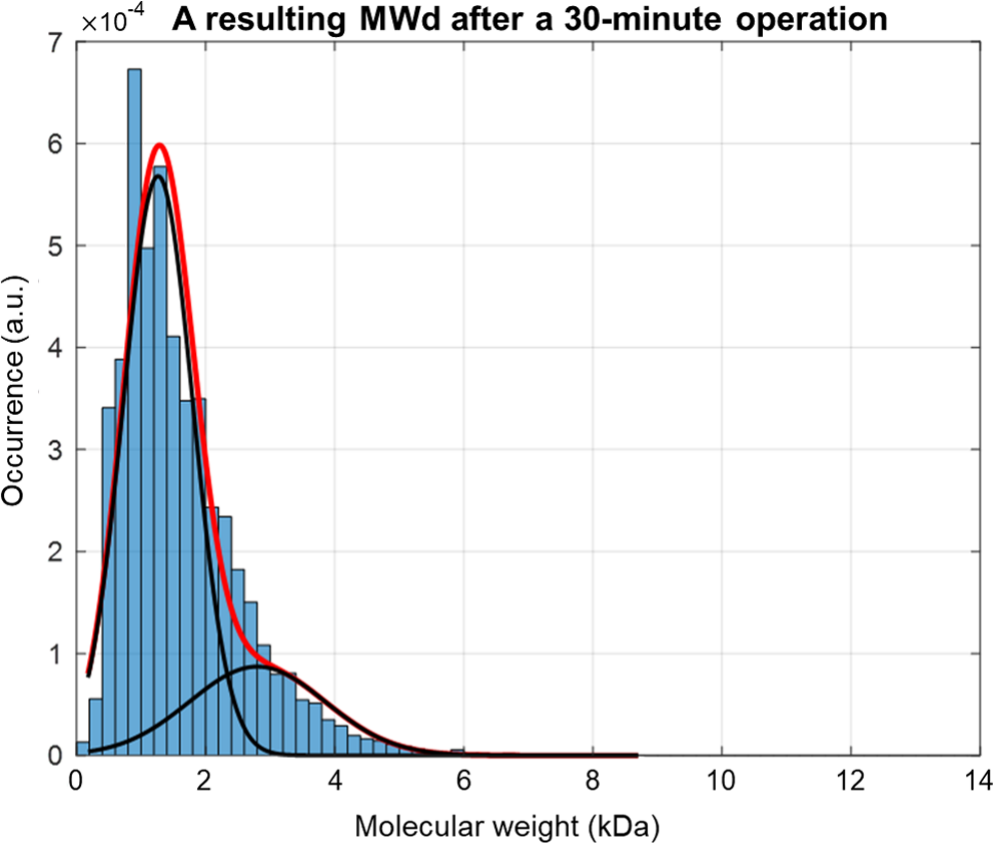
A bimodal lignin MWd after a 30 min operation.

The MPC is formulated as follows
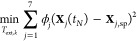
11a
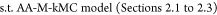
11b
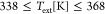
11c
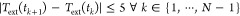
11dwhere ϕ_*j*_ is a weighting constant, **X**_*j*_ stands for the elements to be controlled, *N* represents the number of the prediction horizon, and *T*_ext,*k*_ indicates the external jacket temperature at *t* = *t*_*k*_, respectively. The AA-M-kMC model, integrated as the surrogate model in this MPC formulation, predicts the microscopic reaction rates required to update the system state at each control interval. This allows the MPC to simulate the system efficiently without the computational burden of the full kMC model. By incorporating the surrogate model into the control loop, the MPC optimizes the temperature trajectory based on real-time process feedback, ensuring that key process variables such as MWd and S/G ratio remain within the desired operating ranges. Additionally, this real-time application leverages the reduced computational demand of the AA-M-kMC model, ensuring that the MPC can meet the time constraints required for each sampling interval. As shown in Figure 8, an open-loop operation gives the bimodal lignin MWd after *t* = 30 min, since depolymerization cuts down a lot of chains into smaller fragments. Thus, the target MWd in this framework is set to consist of two peaks in relatively lower and higher ranges. In this regard, **X** is defined as [μ_1_, μ_2_, σ_1_, σ_2_, ω_1_, ω_2_, ρ], where ρ represents the resulting S/G ratio, and the subscript 1 and 2 designate the peak at the lower and higher MW range, respectively. Additionally, the MPC finds the optimal temperature trajectory avoiding a significant rise or drop in the liquor-phase temperature as indicated in eq 11d.

### 3.2 Closed-Loop Operation Results

To demonstrate the performance of the proposed MPC, we carried out a virtual experiment with the original high-fidelity multiscale model. In this work, the control action is taken every 5 min. Also, the set-points are defined as **X**_sp_ = [1000, 2200, 400, 800, 0.70, 0.30, 1.00]. Note that MWd is controlled by the controller, instead of their average values, such as *M*_*n*_ and *M*_w_. For the system to reach the set-points, the *T*_f_ is controlled by varying *T*_ext_.

**Figure 9:**
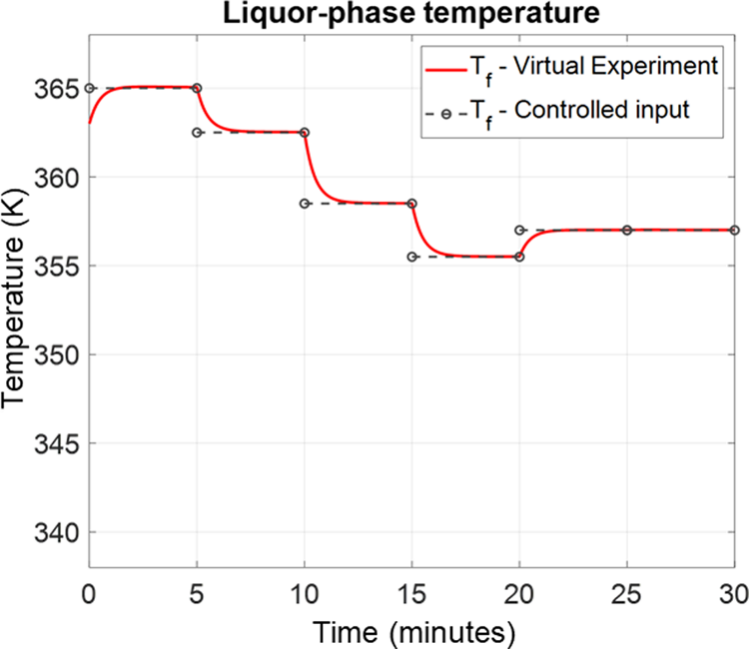
Temperature input trajectory from the proposed MPC framework.

**Figure 10:**
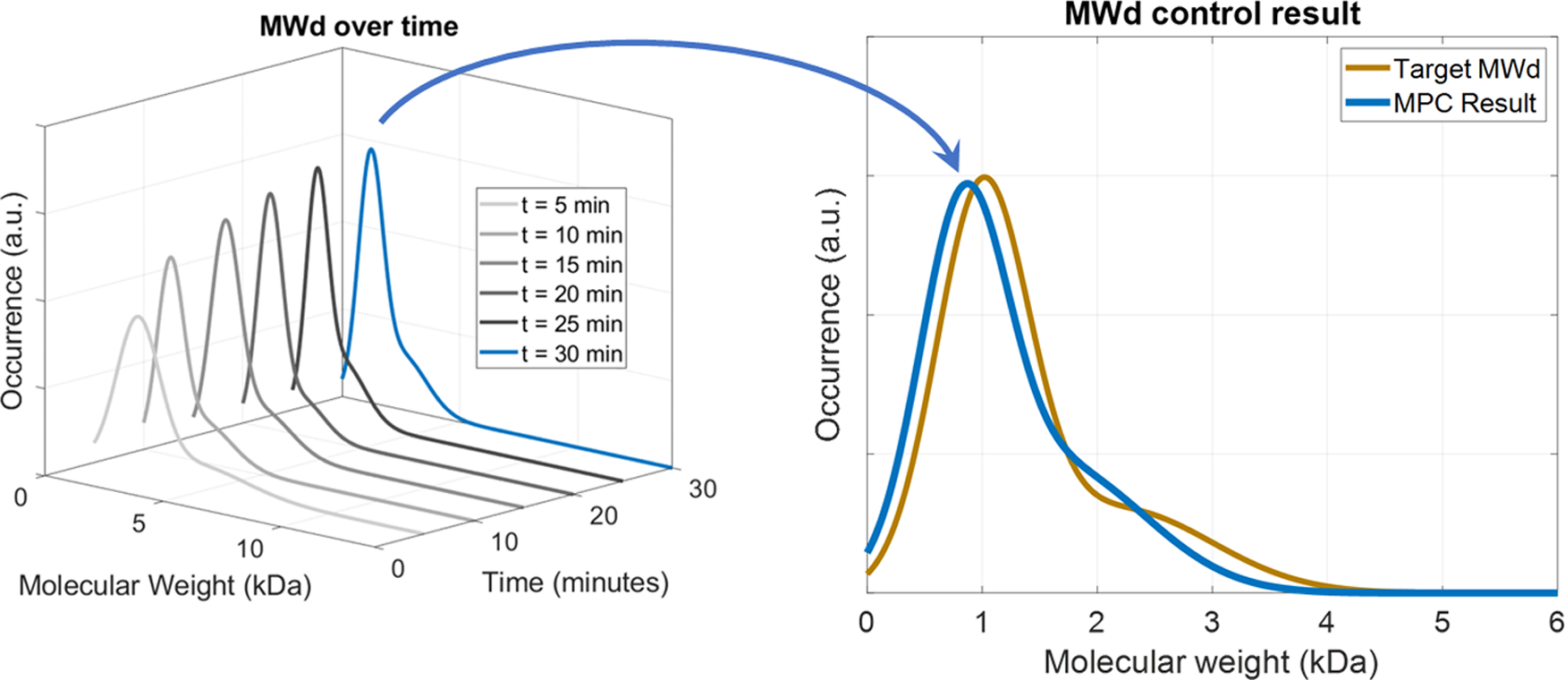
Evolution of lignin MWd compared to the MPC set-points.

The temperature control profile is shown in Figure 9, and the control results are shown in Table 2. As the controller raised the temperature, the delignification became faster. Moreover, the dissolved chains were quickly depolymerized into smaller fragments, resulting in the high lignin population at the low-MW range as shown in Figure 10. Then, the depolymerization becomes slower as the temperature decreases at *t* ≥ 15 min. Consequently, the MWd finally approached the set-point at the end of the operation.

**Table 2:** Set-Points and the Control Results

variable	set-point	closed-loop operation	difference
μ_1_	1.000	0.899	–0.101
σ_1_	0.400	0.425	+0.025
ω_1_	0.700	0.697	–0.003
μ_2_	2.200	1.847	–0.353
σ_2_	0.800	0.773	–0.027
ω_2_	0.300	0.303	+0.003
ρ	1.000	1.012	+0.012

**Figure 11:**
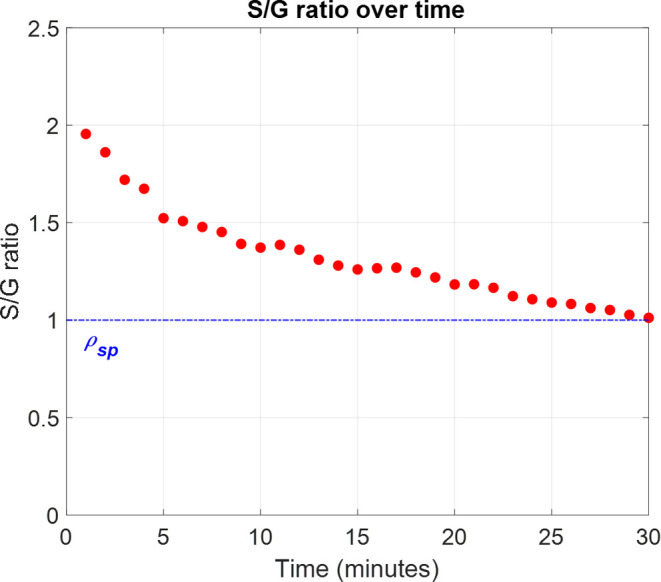
Evolution of the S/G ratio controlled by the proposed MPC.

Another critical property, the S/G ratio, is also regulated through the MPC, as exhibited in Figure 11. Initially, the ratio increased due to the dissolution of S-rich chains, as previously observed in Figure 6b. However, it fell drastically due to the high temperature applied until *t* = 10 min. It seems there were light fluctuations in the S/G ratio for the next 10 min under lower temperatures, but it finally approached the set-point with a low error level of 1.2%.

**Figure 12:**
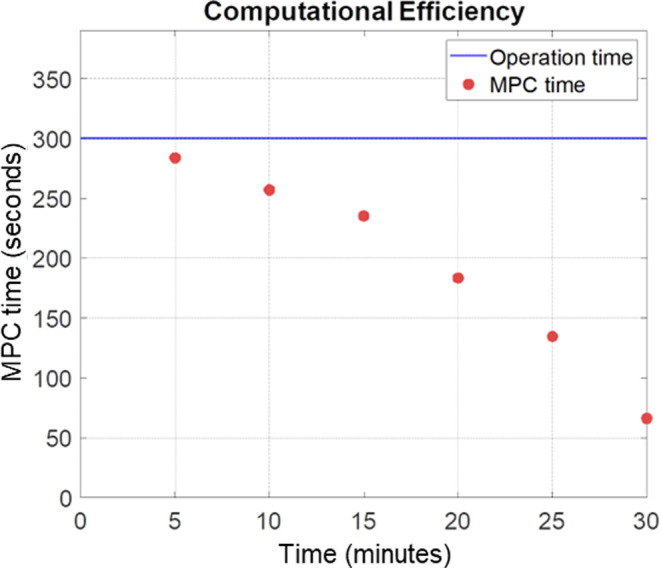
Time required to determine the next temperature for each time segment.

The proposed MPC also enabled real-time control owing to the ANN-assisted acceleration. As highlighted in Figure 12, all the computation times for each segment are less than 5 min, the actual sampling time. Also, while the AA-M-kMC model requires substantially reduced computational demand compared to the existing kMC method, this framework retains the full advantage of the kMC method. In this work, only the rate-calculating step has been replaced with the neural network, while all other procedures, such as the event execution algorithm, remain the same. Therefore, the outputs of the AA-M-kMC model consist of the full information, allowing for detailed system predictions similar to those available from the previous kMC approach.

Hybrid modeling approaches, which integrate first-principles with data-driven models, have been widely employed to enhance model accuracy in complex systems. These methods leverage the strengths of both physics-based and ML models to provide a more comprehensive representation of the system dynamics. In contrast, note that the approach presented in this work focuses primarily on improving computational efficiency rather than solely on enhancing accuracy. By substituting the computationally intensive rate calculation step in the kMC model with ANN predictions, we achieve significant reductions in computational demand while maintaining the detailed system tracking capabilities inherent to the kMC framework. This distinction is important, as our ANN-based acceleration framework not only preserves the accuracy of the original multiscale model but also enables its application in real-time control scenarios where computational resources are constrained. Such an approach complements hybrid modeling efforts by addressing the limitations of computational efficiency in multiscale systems.

While the current framework demonstrates the MPC’s ability to achieve desired lignin properties, future work will explore how disturbance scenarios can be integrated into the surrogate model training. For example, some fluctuations due to feedstock quality or environmental conditions can be handled by integrating observers within the MPC. This could further enhance its resilience to unanticipated process changes, thereby ensuring reliable closed-loop operation in real-world applications.

## 4 Conclusions

In this study, a novel approach to predicting detailed lignin properties in a computationally efficient manner was presented by developing an ANN-accelerated kMC simulation. Specifically, the rate calculation step of the kMC model was replaced with ANN predictions, and the resulting ANN-kMC model performed event selection and system updates in the same manner as the existing kMC algorithm. Therefore, the ANN-kMC model was readily integrated with the macroscopic layer, completing the multiscale model structure, suggested as the AA-M-kMC model in this work. The proposed AA-M-kMC model effectively captured the lignin properties over two phases, according to the system updates from interphase macroscopic events, liquor-phase microscopic events, and their respective governing equations. Moreover, our AA-M-kMC model approach successfully addressed the computational challenges associated with the previous kMC-based model. By replacing the computationally expensive rate calculation step with the ANN predictions, the model significantly reduced the overall simulation time while maintaining the detailed system tracking capability.

Additionally, the AA-M-kMC model was directly implemented in the MPC framework, demonstrating its potential for real-time process control. The virtual experiments, conducted with the high-fidelity kMC model as a benchmark, confirmed the effectiveness of the proposed MPC framework in achieving the target lignin properties, including MWd and S/G ratio. The MPC regulated these properties with high accuracy in real-time, keeping both variables around their respective set-points. To the best of our knowledge, this is the first attempt to incorporate a highly detailed multiscale model directly into the real-time controller. Overall, this work demonstrated a significant advancement in lignin processing methods, offering a powerful tool to enhance the efficiency of biomass fractionation processes.
